# Isolation of Petrocidin A, a New Cytotoxic Cyclic Dipeptide from the Marine Sponge-Derived Bacterium *Streptomyces* sp. SBT348

**DOI:** 10.3390/md15120383

**Published:** 2017-12-06

**Authors:** Cheng Cheng, Eman M. Othman, Helga Stopper, RuAngelie Edrada-Ebel, Ute Hentschel, Usama Ramadan Abdelmohsen

**Affiliations:** 1 Department of Botany II, Julius-von-Sachs Institute for Biological Sciences, University of Würzburg, Julius-von-Sachs-Platz 3, D-97082 Würzburg, Germany; tj.cheng@hotmail.com; 2 Department of Toxicology, University of Würzburg, Versbacher Str. 9, D-97078 Würzburg, Germany; eman@toxi.uni-wuerzburg.de (E.M.O.); stopper@toxi.uni-wuerzburg.de (H.S.); 3 Department of Analytical Chemistry, Faculty of Pharmacy, Minia University, Minia 61519, Egypt; 4 Strathclyde Institute of Pharmacy and Biomedical Sciences, University of Strathclyde, The John Arbuthnott Building, 27 Taylor Street, Glasgow G4 0NR, UK; ruangelie.edrada-ebel@strath.ac.uk; 5 GEOMAR Helmholtz Centre for Ocean Research, RD3 Marine Microbiology, and Christian-Albrechts University of Kiel, Düsternbrooker Weg 20, D-24105 Kiel, Germany; uhentschel@geomar.de; 6 Department of Pharmacognosy, Faculty of Pharmacy, Minia University, Minia 61519, Egypt

**Keywords:** sponges, actinomycetes, streptomyces, cyclic dipeptide, cytotoxic

## Abstract

A new cyclic dipeptide, petrocidin A (**1**), along with three known compounds—2,3-dihydroxybenzoic acid (**2**), 2,3-dihydroxybenzamide (**3**), and maltol (**4**)—were isolated from the solid culture of *Streptomyces* sp. SBT348. The strain *Streptomyces* sp. SBT348 had been prioritized in a strain collection of 64 sponge-associated actinomycetes based on its distinct metabolomic profile using liquid chromatography/high-resolution mass spectrometry (LC-HRMS) and nuclear magnetic resonance (NMR). The absolute configuration of all α-amino acids was determined by HPLC analysis after derivatization with Marfey’s reagent and comparison with commercially available reference amino acids. Structure elucidation was pursued in the presented study by mass spectrometry and NMR spectral data. Petrocidin A (**1**) and 2,3-dihydroxybenzamide (**3**) exhibited significant cytotoxicity towards the human promyelocytic HL-60 and the human colon adenocarcinoma HT-29 cell lines. These results demonstrated the potential of sponge-associated actinomycetes for the discovery of novel and pharmacologically active natural products.

## 1. Introduction

Marine sponges are sources for diverse and novel actinomycetes [1,2,3,4]. A multitude of secondary metabolites [5] have been recovered from marine sponge-associated actinomycetes that display diverse biological activities, such as antimicrobial [6,7,8,9], antiparasitic [10,11], immunomodulatory [12], antichlamydial [13], antioxidant [14], and anticancer [15,16] activities. The conventional natural products isolation scheme has however frequently been challenged owing to the repeated isolation of known compounds, the failure to isolate trace compounds and sometimes the loss of activity during fractionation and compound purification [17]. The lack of detailed chemical information prior to isolation is another draw-back of conventional natural product discovery. By comparison, the metabolomics approach comes with the significant advantage that different active compounds can be determined in crude extracts without comprehensive fractionation or prior isolation of the individual compound while still offering structural information [18,19,20,21]. Our previous study using metabolomics and dereplication tools revealed a chemically distinct strain *Streptomyces* sp. SBT348 by PCA analysis [22]. In the present study, we report on the isolation and structure elucidation of a new cyclic dipeptide, petrocidin A (**1**); three known compounds 2,3-dihydroxybenzoic acid (**2**), 2,3-dihydroxybenzamide (**3**), and maltol (**4**) from strain *Streptomyces* sp. SBT348; as well as the determination of their anti-proliferative properties against HL-60, HT29, and MCF-7 cancer cell lines.

## 2. Results and Discussion

### 2.1. Isolation and Purification of Strain Streptomyces sp. SBT348

**Figure 1:**
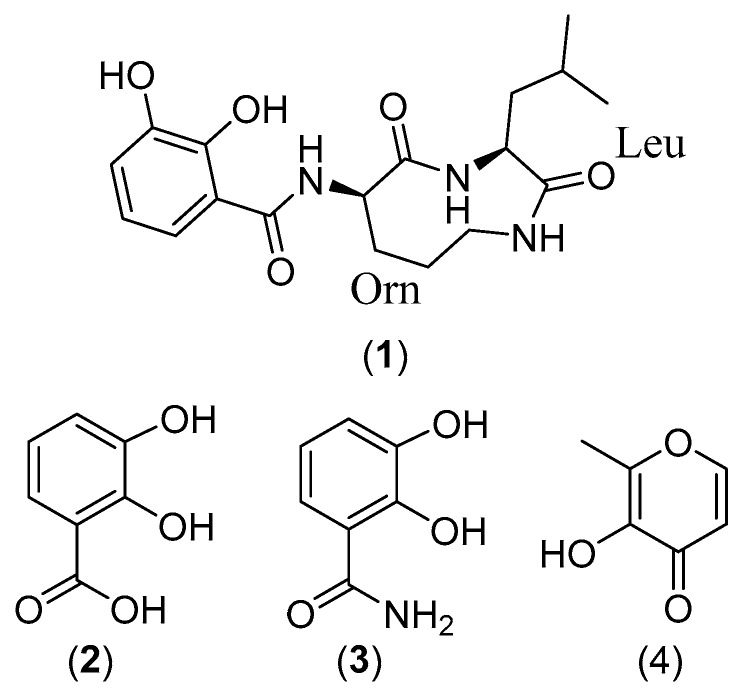
Compounds isolated from outlying strain *Streptomyces* sp. SBT348; petrocidin A (**1**), 2,3-dihydroxybenzoic acid (**2**), 2,3-dihydroxybenzamide (**3**), and maltol (**4**).

*Streptomyces* sp. SBT348 (GenBank accession No. KP238417) was cultivated from the Mediterranean sponge *Petrosia ficiformis* that was collected from Milos, Greece [22]. Our study using metabolomics and dereplication tools prioritized the strain *Streptomyces* sp. SBT348 based on its chemical uniqueness by Principal Component Analysis (PCA) (Figure 1). The ethyl acetate extract of the solid culture of *Streptomyces* sp. SBT348 was fractionated by preparative HPLC to afford a new cyclic dipeptide (**1**), and three known compounds-2,3-dihydroxybenzoic acid (**2**) [23], 2,3-dihydroxybenzamide (**3**) [24], and maltol (**4**) [25]. The structures of compounds **2**, **3**, and **4** were determined based on the MS and NMR elucidation, as well as comparison to the literature and Integrated Spectral Database System of Organic Compounds developed by AIST (National Institute of Advanced Industrial Science and Technology). The structure elucidation of the new cyclic dipeptide compound **1** was discussed as follows.

### 2.2. Structure Elucidation

**Figure 2:**
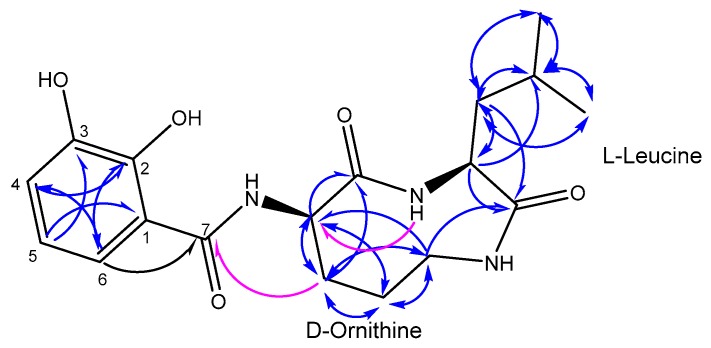
Structure of petrocidin A (**1**) with HMBC key correlations (blue, arrows from H to C in MeOD-*d*_4_; Purple, key arrows from H to C in DMSO-*d*_6_).

Compound **1** (1.8 mg, yield 0.49%) was purified as a colorless solid. The following NMR spectral data were acquired using a 600 MHz instrument: ^1^H, ^13^C, ^13^C-DEPT135 (Distortionless Enhancement by Polarization Transfer), ^1^H-^1^H COSY (correlation spectroscopy), ^1^H-^1^H NOESY (Nuclear Overhauser effect spectroscopy), ^1^H-^13^C HSQC (heteronuclear single quantum correlation), and ^1^H-^13^C HMBC (Heteronuclear Multiple Bond Correlation) (optimized to *J* = 8.3 Hz) in MeOD-*d*_4_, as well as ^1^H-^13^C HMBC (optimized to *J* = 8.3 Hz) in DMSO-*d*_6_ and tabulated in Table 1. The molecular formula was determined to be C_18_H_25_N_3_O_5_ (*m*/*z* 386.1692 [M + Na]^+^ calcd. for C_18_H_25_N_3_O_5_Na) from its HRESI-MS (High-Resolution Electrospray Ionization Mass Spectrometry) indicating eight degrees of unsaturation. The ^1^H spectrum displayed three aromatic protons at δ_H_ 6.98 (1H, d, *J* = 7.8 Hz), 6.71 (1H, t, *J* = 7.8, 15.8 Hz), and 7.34 (1H, d, *J* = 7.8 Hz) ppm, three methine protons at δ_H_ 1.89 (1H, m), 4.13 (1H, t, *J* = 5.6, 11.4 Hz), and 4.26 (1H, t, *J* = 8.5, 16.1 Hz), eight methylene protons at δ_H_ 3.51 (2H, t, *J* = 4.7, 8.9 Hz), 2.30 (1H, m), 2.02 (1H, m), 2.01 (1H, m), 1.94 (1H, m), 1.91 (1H, m), and 1.52 (1H, m) ppm, and six methyl protons at δ_H_ 0.96 (3H, d, *J* = 3.7 Hz) and 0.95 (3H, d, *J* = 3.7 Hz) ppm. ^13^C, ^3^C-DEPT135 and HSQC spectra displayed six aromatic carbons at δ_C_ 114.7, 119.6, 121.3, 121.9, 147.0, and 151.7 ppm; three ester/amide-type carbonyls at δ_C_ 168.3, 172.8, and 174.2 ppm; three methine carbons at δ_C_ 60.3, 54.6, and 25.8 ppm; four methylene carbons at δ_C_ 23.7, 29.1, 39.4, and 46.4 ppm; and two methyl carbons at δ_C_ 22.2 and 23.2 ppm. The three aromatic protons at δ_H_ 6.98, 6.71, and 7.34 ppm were assigned to H-4, H-5, and H-6, respectively by COSY spectrum and indicated an adjacent three-substitution aromatic system as part of the structure. The deshielded carbon resonances at δ_C_ 151.7 and 147.0 ppm suggested two hydroxyl substitutions on the aromatic ring, and were assigned to C-2 and C-3 respectively, with the correlations observed amongst H-4, H-5, and H-6 in the HMBC spectrum (Figure 2). The other aromatic carbon resonances at δ_C_ 114.7, 121.3, 119.6, 121.9 ppm were attributed to C-1, C-4, C-5, C-6, respectively according to the cross-peaks observed in the HSQC and HMBC spectra. The strong correlation observed in the HMBC spectrum between H-6 and one of the ester/amide-type carbonyls at δ_C_ 174.2 ppm has assigned δ_C_ 174.2 to C-7 (Figure 2). The HSQC and HMBC spectra also exhibited standard resonance signals for amino acids by the observation of two α-methines signals at δ_H_ 4.26 (1H, t, *J =* 8.5, 16.1 Hz), 4.13 (1H, t, *J =* 5.6, 11.4 Hz) with their connected deshielded carbons at δ_C_ 60.3 and 54.6 ppm, respectively, as well as HMBC cross-peaks between the α-protons and ester/amide-type carbonyls at δ_C_ 172.8 and 168.9 ppm (Figure 2). Analysis of the COSY, HSQC, and HMBC data, as well as degrees of unsaturation assigned one ornithine and one leucine moiety presented in a cyclic nature. The connection between the aromatic part and the cyclic peptide moiety was confirmed by the correlation displayed in HMBC (using DMSO-*d*_6_ as solvent) between Orn β-H and C-7 (Figure 2). The proton and carbon resonances were also compared to the human metabolites database (HMDB) with ornithine and leucine individually, as well as 2,3-dihydroxybenzamide-scaffold with compound **2** and literature [24], and they showed consistent results. The absolute configuration of leucine and ornithine was determined by acid hydrolysis followed by Marfey’s derivatization and comparison to their analogues of the authentic amino acids by HPLC analysis. The Marfey’s analysis of compound **1** revealed the absolute configuration of leucine to be l and ornithine to be d-configuration. Compound **1** was identified as 2,3-dihydroxy-N-((3S,6R)-3-isobutyl-2,5-dioxo-1,4-diazonan-6-yl) benzamide, a new cyclic dipeptide, and given the name petrocidin A.

Compounds **2**, **3**, and **4** were isolated as colorless solid from fraction 1, 2, and 5 respectively. The molecular formula was established as C_7_H_6_O_4_ (found at EI *m*/*z* 154.0, calcd. 154.0266) for compound **2**, C_7_H_7_NO_3_ (found at EI *m*/*z* 153.0, calcd. 153.0426) for compound **3**, and C_6_H_6_O_3_ (found at EI *m*/*z* 126.0, calcd. 126.0317) for compound **4**, respectively. An exact mass search in the Database of Natural Products (2015) and comparison of the spectral data with the literature determined compound **2** as 2,3-dihydroxybenzoic acid [26], compound **3** as 2,3-dihydroxybenzamide [24], and compound **4** as Maltol [27].

### 2.3. Biological Activities of Isolated Compounds

Compounds 2,3-dihydroxybenzoic acid (**2**) and 2,3-dihydroxybenzamide (**3**) were both previously isolated from a marine alga-derived actinomycete strain USF-TC31 and exhibited potent antioxidant activity in DPPH (2,2-Diphenyl-1-picrylhydrazyl) radical scavenging assay [24]. 2,3-dihydroxybenzoic acid (**2**) was also identified as the precursor of a group of catecholate siderophores which were described from *Vibrio* sp. and *Salmonella* sp. embedding with the substructure of 2,3-dihydroxybenzamide [27,28,29,30,31]. The new compound petrocidin A (**1**) enclosing the moiety of 2,3-dihydroxybenzamide as part of the structure indicated that 2,3-dihydroxybenzoic acid (**2**) might be the direct precursor of petrocidin A (**1**). Maltol (**4**) was previously reported from different plants [24,32,33], but was also isolated as a bacterial metabolite from *Streptomyces* sp. GW3/1538 [34].

Microsomal prostaglandin E_2_ synthase-1 (mPGES-1) has been reported to be over-expressed in a group of tumor cells-such as HL-60, HT-29, and MCF-7 cell lines-but not in normal tissues [35,36]. The moiety of 2,3-dihydroxybenzamide was proposed as an active scaffold in the search for new mPGES-1 inhibitors used in anti-inflammatory and anticancer process [37]. The anti-proliferative properties of the four compounds were evaluated against the three cancer cell lines including the HL-60, HT-29, and MCF-7 cell lines. Petrocidin A (**1**) displayed significant cytotoxic effects towards HL-60 and HT-29 cells with the IC_50_ values of 3.9 and 5.3 μg/mL, respectively. While, 2,3-Dihydroxybenzamide (**3**) exhibited potent cytotoxicity towards the same two cell lines with the IC_50_ values of 5.5, and 3.8 μg/mL, respectively. 2,3-Dihydroxybenzoic acid (**2**) and maltol (**4**) did not exhibit any significant cytotoxicity at the examined concentrations. These results suggested that the new cyclic dipeptide petrocidin A (**1**) and 2,3-dihydroxybenzamide (**3**) which is a part of the peptidic structure exhibiting anti-proliferative effects towards HL-60 and HT29 cancer cell lines which might be as mPGES-1 inhibitors.

## 3. Materials and Methods

### 3.1. Cell Lines, Chemicals, and Biochemical

HL-60 cells (human promyelocytic cell) were grown in 5% (*v*/*v*) CO_2_ in RPMI 1640 medium at 37 °C, supplemented with 10% (*v*/*v*) 1% (*w*/*v*) l-glutamine, and 0.4% (*w*/*v*) antibiotics (50 U/mL penicillin and 50 mg/mL streptomycin). The HT-29 cells (human colon adenocarcinoma cell) were cultured at 37 °C, 5% (*v*/*v*) CO_2_ in Dulbecco’s modified Eagle medium (DMEM) with high glucose (4.5 g/L) supplemented with 10% (*v*/*v*) fetal bovine serum (FBS), 1% (*w*/*v*) l-glutamine, and 0.4% (*w*/*v*) antibiotics (50 U/mL penicillin, 50 mg/mL streptomycin). MCF-7 cells (Human breast adenocarcinoma cell) were cultured at 37 °C, 5% (*v*/*v*) CO_2_ in RPMI1640 medium, supplemented with 5% (*v*/*v*) fetal bovine serum (FBS), 1% (*w*/*v*) l-glutamine, 1% sodium pyruvate and 0.4% (*w*/*v*) antibiotics (50 U/mL penicillin, 50 mg/mL streptomycin). Cells were obtained from the American Type Culture Collection (ATCC, Rockville, MD, USA; HPACC, Salisbury, UK) and routinely sub-cultured twice per week. All chemicals and reagents were purchased from Sigma Aldrich (Darmstadt, Germany).

### 3.2. Bacteria

*Streptomyces* sp. SBT348 (GeneBank accession No. KP238417) was recovered from the Mediterranean sponge *Petrosia ficiformis* which was collected by SCUBA diving from offshore Pollonia, Milos, Greece (36.76612° N; 24.51530° E) at 5–7 m depth in May 2013.

### 3.3. Large-Scale Fermentation, Extraction, and Isolation

Three hundred ISP2 agar plates (square 120 × 120 mm), each inoculated with 100 μL of five days liquid culture of *Streptomyces* sp. SBT348 respectively were incubated at 30 °C for seven days. The agar media with bacterial biomass were scalped into small pieces and transferred to 1 L Erlenmeyer flasks. Five hundred mL of ethyl acetate/flask were added to submerge the agar pieces and macerated the medium culture under shaking at 175 rpm for overnight. The macerations were subsequently filtered by gravity using filter paper (A. Hartenstein, Würzburg, Germany). The filtrates were combined and evaporated by under vacuum (Büchi, Essen, Germany). Three-hundred and seventy mg of the dried crude EtOAc extract obtained from the solid culture of strain *Streptomyces* sp. SBT348 was fractionated by semi-preparative HPLC (Agilent, Waldbronn, Germany) using H_2_O/ACN (95%:5%) initially for 5 min, then by a linear gradient to 100% ACN within 40 min, which was followed by an isocratic condition of 100% ACN for a further 5 min on the Prep C18 column (5 μm, 10 × 250 mm, (Waters XBridge, Eschborn, Germany), with a flow rate of 3.0 mL/min to yield five fractions. Compounds **1**–**4** were purified from peaks rich fraction Nr. 3 by semi-preparative HPLC using Onyx Monolithic semi-prep RP-C18 column (5 μm, 10 × 100 mm, Phenomenex, Aschaffenburg, Germany).

### 3.4. LC-MS Analysis

Accurate electrospray ionization mass spectra (ESI) were obtained by a Synapt G2 HDMS qTOF-Mass Spectrometer (Waters, Eschborn, Germany). The ESI was operated in the positive and negative ionization modes. The capillary voltage was set to 0.8 kV and nitrogen (at 350 °C, the flow rate of 800 L/h) was used as desolvation gas. The molecular ion was determined by quadrupole in a wide-band RF mode, and data was acquired over the mass range of 50–1200 Da. Product ion scan was optimized for different analyses using different collision voltage from 10 to 25 eV. MassLynx (version 4.1, Waters, Bremen, Germany) was utilized to acquire and process mass spectrum.

### 3.5. Marfey’s Analysis

The absolute configuration of α-H that presented in compound **4** was performed by Marfey’s derivatization and compared to the purchased amino acid with d and l configurations (Sigma, Darmstadt, Germany) by HPLC. Compound **4** (0.8 mg) was initially hydrolyzed with 6 M HCl (2 mL) in the water bath at 100 °C for 24 h. The hydrolysate was cooled to room temperature, dried using vacuum evaporator, and finally dissolved in 100 μL of water. The Marfey’s derivatization was carried out by adding 100 μL of 1% Marfey’s reagent (1-Fluoro-2,4-dinitrophenyl-5-l-alanine amid) dissolved in acetone and 20 μL of 1 M NaHCO_3_ (H_2_O) to 50 μL of the hydrolysate of compound **4**, as well as 50 mM standard amino acid (d-Leu, l-Leu, d-Orn, and l-Orn) respectively, and incubated at 40 °C for 1 h with frequent shaking. The reaction was stopped by adding 10 μL of 2 M HCl after cooling. The Marfey’s derivatization products were finally dried and prepared in MeOH for further HPLC analysis. The HPLC chromatography was carried out on Gemini-NX RP-C18 column (Phenomenex, Aschaffenburg, Germany by eluting with H_2_O/CH_3_CN (95%:5%) for the first 5 min, linearly gradient to 100% CH_3_CN within 25 min, and held at 100% CH_3_CN for a further 5 min with a flow rate at 1 mL/min and UV detection at 340 nm. The configuration was eventually determined with the observation of the same retention times compared to the purchased standard enantiomeric amino acids (Bhushan and Bruckner, 2004; Kochhar and Christen, 1989; Marfey, 1984).

### 3.6. MTT ((3-(4,5-Dimethylthiazol-2-yl)-2,5-diphenyltetrazolium bromide)) Assay

Cell proliferation was evaluated in cell lines by the MTT assay in triplicates. Briefly, cells were plated in a 96-well microtiter plate at a density of 1 × 10^4^ cells per well in a final volume of 100 μL of culture medium. These cells were treated for 24 h with tested compound at 37 °C with 5% CO_2_. After treatment, the cells were immediately incubated with 10 μL MTT (5.0 mg/mL) for 4 h at 37 °C. The cells were lysed in 100 μL of lysis buffer (isopropanol, conc. HCl and Triton X-100) for 10 min at room temperature and 300 rpm/min shaking. The enzymatic reduction of MTT to formazan crystals that dissolved in DMSO was quantified by photometry at 570 nm. Dose-response curves were generated and the IC_50_ values were defined as the concentration of compound required to inhibit cell proliferation by 50%. 5-Flurouracil was used as a positive control.

### 3.7. Compounds Characterization

#### 3.7.1. Petrocidin A (**1**)

Colorless solid (1.8 mg, *R*_t_ = 14.72 min); UV (EtOH) *λ*_max_ 220, 320 nm; IR (KBr) *ν*_max_ 4376, 4203, 3353, 1859, 1616, 1500, 1204 cm^−1^; ^1^H NMR (MeOD-*d*_4_, 600 MHz,): δ = 6.98 (1H, d, *J* = 7.8 Hz, H-4), 6.71 (1H, t, *J* = 7.8 Hz, H-5), 7.23 (1H, d, *J* = 7.8 Hz, H-6), 4.26 (1H, t, *J* = 8.5 Hz, Orn α-proton), 2.30 (1H, m, Orn β-proton), 2.01 (1H, m, Orn β-proton), 2.02 (1H, m, Orn γ-proton), 1.94 (1H, m, Orn γ-proton), 3.51 (2H, t, *J* = 4.7 Hz, Orn δ-proton), 4.13 (1H, t, *J* = 5.6 Hz, Leu α-proton), 1.52 (1H, m, Leu β-proton), 1.91 (1H, m, Leu β-proton), 1.89 (1H, m, Leu γ-proton), 0.96 (3H, d, *J* = 3.7 Hz, Leu δ-proton), 0.95 (3H, d, *J* = 3.7 Hz, Leu δ-proton) ppm. ^1^H NMR (DMSO-*d*_6_, 600 MHz,): 8.02 (1H, s, Leu NH). ^13^C NMR (MeOD-*d*_4_, 150 MHz,): δ = 114.7 (C, C-1), 151.7 (C, C-2), 147.0 (C, C-3), 121.3 (CH, C-4), 119.6 (CH, C-5), 121.9 (CH, C-6), 174.2 (C, C-7), 172.8 (C, Orn CO), 60.3 (1C, Orn α-carbon), 29.1 (1C, Orn β-carbon), 23.7 (1C, Orn γ-carbon), 46.4 (1C, Orn δ-carbon), 168.9 (1C, Leu CO), 54.6 (1C, Leu α-carbon), 39.4 (1C, β-carbon), 25.8 (1C, γ-carbon), 22.2 (1C, δ-carbon), 23.2 (1C, δ-carbon). ESI-HRMS *m*/*z* 383.1692 [M + Na]^+^, C_18_H_25_N_3_O_5_Na (calcd. 383.1691); Anal. Calcd. for C_18_H_25_N_3_O_5_Na: C, 59.49; H, 6.93; N, 11.56; O, 22.01.

#### 3.7.2. 2,3-Dihydroxybenzoic Acid (**2**)

White solid (2.7 mg, *R*_t_ = 12.30 min); UV (EtOH) *λ*_max_ 254, 320 nm; IR (KBr) *ν*_max_ 3664, 1980, 1506, 1035 cm^−1^; ^1^H NMR (MeOD-*d*_4_, 600 MHz,): δ = 6.95 (1H, dd, *J* = 7.9, 1.5 Hz, H-4), 6.71 (1H, t, *J* = 8.3 Hz, H-5), 7.24 (3H, dd, *J* = 7.9, 1.5 Hz, H-6); ^13^C NMR (MeOD-*d*_4_, 150 MHz,): δ = 115.9 (C, C-1), 151.1 (C, C-2), 147.3 (C, C-3), 120.0 (CH, C-4), 119.5 (CH, C-5), 119.4 (CH, C-6), 174.5 (C, C-7); EI 153.0 Dalton, C_7_H_7_NO_3_ (calcd. 153.0426); Anal. Calcd. for C_7_H_7_NO_3_: C, 54.90; H, 4.61; N, 9.15; O, 34.31.

#### 3.7.3. 2,3-Dihydroxybenzamide (**3**)

White soild (2.3 mg, *R*_t_ = 9.94 min); UV (EtOH) *λ*_max_ 247, 310 nm; IR (KBr) *ν*_max_ 3571, 2014, 1868, 1458, 1227 cm^−1^; ^1^H NMR (DMSO-*d*_6_, 600 MHz,): δ = 6.98 (1H, dd, *J* = 7.8, 1.6 Hz, H-4), 6.71 (1H, t, *J* = 7.8 Hz, H-5), 7.24 (1H, dd, *J* = 7.8, 1.6 Hz, H-6); ^13^C NMR (DMSO-*d*_6_, 150 MHz,): δ = 113.5 (C, C-1), 150.5 (C, C-2), 145.9 (C, C-3), 120.5 (CH, C-4), 118.4 (CH, C-5), 120.0 (CH, C-6), 172.5 (C, C-7); EI 154.0 Dalton, C_7_H_6_O_4_ (calcd. 154.0266); Anal. Calcd. for C_7_H_6_O_4_: C, 54.55; H, 3.92; O, 41.52.

#### 3.7.4. Maltol (**4**)

Colorless solid (1.6 mg, *R*_t_ = 11.72 min ); UV (EtOH) *λ*_max_ 220, 284 nm; IR (KBr) *ν*_max_ 3928, 1936, 1313, 293 cm^−1^; ^1^H NMR (MeOD-*d*_4_, 600 MHz,): δ = 6.38 (1H, d, *J* = 5.5 Hz, H-5), 7.94 (1H, d, *J* = 5.5 Hz, H-6), 2.35 (3H, s, H-7); ^13^C NMR (MeOD-*d*_4_, 150 MHz,): δ = 152.2 (C, C-2), 144.6 (C, C-3), 175.3 (C, C-4), 114.4 (CH, C-5), 156.3 (CH, C-6), 14.2 (CH3, C-7); EI 126.0 Dalton, C_6_H_6_O_3_ (calcd. 126.0317); Anal. Calcd. for C_6_H_6_O_3_: C, 57.14; H, 4.80; O, 38.06.

## 4. Conclusions

In summary, four compounds including a new cyclic dipeptide, petrocidin A (**1**), and three known compounds-2,3-dihydroxybenzoic acid (**2**), 2,3-dihydroxybenzamide (**3**), and maltol (**4**)-were isolated from the solid culture of *Streptomyces* sp. SBT348 which was derived from marine sponge *Petrosia ficiformis*. The new cyclic dipeptide petrocidin A (**1**) and the known 2,3-dihydroxybenzamide (**3**) displayed cytotoxicity against HL-60 and HT-29 cancer cell lines. These results demonstrated that sponge-associated actinomycetes are a promising source for the discovery of novel and pharmacologically active natural products.

## Figures and Tables

**Table 1:** NMR-spectroscopic data of petrocidin A (**1**) in MeOD-*d*_4_ and DMSO-*d*_6_ (1H: 600 MHz; ^13^C: 150 MHz).

Position	δ_C,_ Type	δ_H_ (*J*, Hz)	COSY	HMBC ^a^	HMBC ^b^
1	114.7, C	-	-	-	-
2	151.7, C	-	-	-	-
3	147.0, C	-	-	-	-
4	121.3, CH	6.98 (1H, d, *J* = 7.8 Hz)	6.71	151.7, 147.0, 121.9	-
5	119.6, CH	6.71 (1H, t, *J* = 7.8 Hz)	6.98, 7.34	147.0, 114.7, 121.3	-
6	121.9, CH	7.34 (1H, d, *J* = 7.8 Hz)	6.71	174.2, 151.7, 121.3	-
7	174.2, C	-	-	-	-
Orn	-	-	-	-	-
CO	172.8, C	-	-	-	-
α	60.3, CH	4.26 (1H, t, *J* = 8.5 Hz)	2.30, 2.01	172.8, 29.1, 23.7	-
β	29.1, CH_2_	2.30 (1H, m), 2.01 (1H, m)	2.02, 1.94	46.4, 23.7, 60.3, 172.8	171.9 (C-7)
γ	23.7, CH_2_	2.02 (1H, m), 1.94 (1H, m)	2.30, 2.01, 2.02, 1.94	29.1, 60.3, 46.4	-
δ	46.4, CH_2_	3.51 (2H, t, *J* = 4.7 Hz)	2.02, 1.94	168.9, 29.1, 23.7	-
Leu	-	-	-	-	-
NH	-	8.02 (1H, s, DMSO-*d*_6_)	-	-	37.8 (Leu β-C), 52.6 (Leu α-C), 58.5 (Orn α-C), 166.6 (Leu CO)
CO	168.9, C	-	-	-	-
α	54.6, CH	4.13 (1H, t, *J* = 5.6 Hz)	1.91, 1.52	168.9, 39.4, 25.8	-
β	39.4, CH_2_	1.52 (1H, m), 1.91 (1H, m)	1.89, 4.13	168.9, 54.6, 25.8, 23.2, 22.2	-
γ	25.8, CH	1.89 (1H, m)	0.96, 0.95, 1.52, 1.91	39.4	-
δ	22.2, 23.2, CH_3_	0.96 (3H, d, *J* = 6.5 Hz)	1.89	25.8, 39.4	-
		0.95 (3H, d, *J =* 3.1, 6.2 Hz)			

**^a^** HMBC correlations, optimized for 8.3 Hz in MeOD-*d*_4_, are from protons stated to the indicated carbon; **^b^** HMBC correlations, optimized for 8.3 Hz in DMSO-*d*_6_, are from protons stated to the indicated carbon.
